# Gamma Tocopherol Reduced Chemotherapeutic-Induced ROS in an Ovarian Granulosa Cell Line, But Not in Breast Cancer Cell Lines In Vitro

**DOI:** 10.3390/antiox9010051

**Published:** 2020-01-07

**Authors:** Daniela Figueroa Gonzalez, Fiona Young

**Affiliations:** Medical Biotechnology, College of Medicine and Public Health, Flinders University, Adelaide 5042, Australia; daniela.figueroagonzalez@flinders.edu.au

**Keywords:** breast cancer, reactive oxygen species, tocopherol, doxorubicin, cyclophosphamide, ovary, granulosa cell, infertility

## Abstract

Doxorubicin and cyclophosphamide are used to treat breast cancer, but they also cause infertility through off-target cytotoxicity towards proliferating granulosa cells that surround eggs. Each chemotherapeutic generates reactive oxygen species (ROS) but the effects of the combination, or the antioxidants alpha (αToc) and gamma tocopherol (γToc) on ROS in breast cancer or ovarian cells are unknown. Human breast cancer (MCF7, T47D) and ovarian cancer (OVCAR, COV434) cells were loaded with DCDFA and exposed (1, 2, 3, 24 h) to the MCF7-derived EC25 values of individual agents, or to combinations of these. ROS were quantified and viable cells enumerated using crystal violet or DAPI. Each chemotherapeutic killed ~25% of MCF7, T47D and OVCAR cells, but 57 ± 2% (doxorubicin) and 66 ± 2% (cyclophosphamide) of the COV434 granulosa cells. The combined chemotherapeutics decreased COV434 cell viability to 34 ± 5% of control whereas doxorubicin + cyclophosphamide + γToc reduced ROS within 3 h (*p* < 0.01) and reduced cytotoxicity to 54 ± 4% (*p* < 0.05). αToc was not cytotoxic, whereas γToc killed ~25% of the breast cancer but none of the ovarian cells. Adding γToc to the combined chemotherapeutics did not change ROS or cytotoxicity in MCF7, T47D or OVCAR cells. The protection γToc afforded COV434 granulosa cells against chemotherapy-induced ROS and cytotoxicity suggests potential for fertility preservation.

## 1. Introduction

Intracellular reactive oxygen species (ROS) [1,2,3] are crucial for normal cell metabolism [3,4,5,6] and ROS generation is highly regulated by either enzymatic (catalases, peroxidases and dismutases) or non-enzymatic (vitamin A, C or E) reductive molecules. Disturbances in cellular redox balance can lead to an over-accumulation of ROS [1]. Cells also produce ROS after exposure to radiation or chemotherapeutics [7], many of which induce ROS to toxic levels as part of their mechanism of action [8].

The combination of doxorubicin (Dox) and cyclophosphamide is often used to treat breast cancer [9,10,11]. Dox is an anthracycline agent that causes apoptosis by intercalating into double-stranded DNA and inhibiting topoisomerase-II [12]. A second mechanism of action involving ROS has also been described [5,13,14].

Cyclophosphamide is an alkylating agent that requires hepatic metabolic activation [15], which generates 4-hydroxycyclophosphamide and aldophosphamide [16]. Aldophosphamide is metabolized into phosphoramide mustard and acrolein, which increases ROS production in a variety of cell lines [17,18].

Dox and cyclophosphamide are associated with a variety of adverse effects in vivo, including T-cell suppression, chronic cardiotoxicity and premature ovarian failure [19,20,21,22,23,24,25,26,27,28]. It has been proposed that chemotherapeutics cause premature ovarian failure by targeting proliferating granulosa cells in growing follicles [22,23]. Since granulosa cells synthesise anti-Müllerian hormone (AMH), the loss of follicles due to chemotherapeutic-induced cytotoxicity causes a consequent depletion in circulating AMH, which results in the activation and recruitment of dormant primordial follicles into the growing pool [23]. It is thought that the in vivo administration of repeated cycles of Dox and cyclophosphamide diminishes the cohort of active growing follicles, reduces the reserve of primordial follicles, and results in premature ovarian failure [21,23,24,25,26,27,28,29]. 

Although the toxicity of cyclophosphamide and Dox (as single agents) on the ovary is well established [21,23,24,25,26,27,28,29] there are no reports that describe the effect of the combination of Dox and cyclophosphamide on proliferating granulosa cells.

The tocopherols (alpha, beta, gamma and delta) and tocotrienols (alpha, beta, gamma and delta) that together form Vitamin E [30] act as free radical scavengers in cell membranes [31]. α-tocopherol (αToc) is the most abundant form in nature, while γ-Tocopherol (γToc) is the most common form in the human diet [31]. It has been proposed that Dox-induced cardiotoxicity is the result of ROS-induced membrane lipid peroxidation [32], and vitamin E deficiency results in histological features that are comparable to Dox-treated cardiac tissue [19,32]. Although the administration of αToc to breast cancer patients before chemotherapy elevated serum concentrations 8-fold, there were no other observable effects [19]. However, the effects of αToc on post-chemotherapeutic ovarian function were not examined. γToc delayed the formation of breast cancer tumours in rodent models [33], induced apoptosis in breast cancer cells in vitro [34,35] and may prevent breast cancer in vivo [36,37]. Additionally, a mixture of γ and delta tocopherol down-regulated the expression of estrogen receptor and inhibited estradiol-induced human MCF-7 breast cancer cell proliferation in vitro [36]. 

Both α and γ tocopherol are antioxidants with the potential to reduce chemotherapeutic-induced ROS damage, and consequently reduce premature ovarian failure. Reduced ROS, however, could also lead to decreased efficacy against breast cancer cells. γToc has both reductive power and anticancer activity [33], and this led to our hypothesis that gamma tocopherol, but not alpha tocopherol, would augment the cytotoxic activity of the combination of Dox and cyclophosphamide against breast cancer cells in vitro, whilst simultaneously reducing ROS generation.

## 2. Materials and Methods

### 2.1. Chemicals and Reagents

All chemicals and reagents used in the study were obtained from Sigma-Aldrich (Sydney, Australia), unless otherwise specified. The 2′,7′-dichlorofluorescin diacetate (DCFDA) cellular ROS detection assay kit was purchased from Abcam (Melbourne, Australia). 

### 2.2. Preparation of Solutions

Supplemented RPMI was prepared by mixing 500 mL of phenol red-free RPMI with foetal calf serum (FCS, DKSH, Victoria, Australia) at 10% for MCF-7 and T47D, and 20% for OVCAR-3 cells, and 1% *v*/*v* of 10,000 unit/mL penicillin + 10 mg/mL streptomycin (pen-strep). Supplemented RPMI with 20% FCS also contained 5 μg/mL of recombinant human insulin for use with OVCAR-3 cells. Supplemented DMEM/F-12 was prepared by mixing phenol red-free DMEM/F-12, 10% FCS and 1% *v*/*v* of pen-strep. A total of 10 mL Hank’s balanced salt solution (HBSS, provided by the DCFDA ROS assay kit manufacturer) was added to 90 mL ddH_2_O. DCFDA was diluted in 1X HBSS to generate a solution of 10 μM. The DCFDA ROS assay positive control, ter-butyl hydrogen peroxide (TBHP), was diluted in supplemented media (RPMI or DMEM/F12) without phenol red, to give final concentrations of 12.5 and 50 μM. Stock solutions of 100 µM Dox and 1000 µM 4-hydroperoxycyclophosphamide (4-Cyc, ThermoFisher Scientific, Victoria, Australia) were prepared in supplemented media (RPMI or DMEM/F-12) and kept at 4 and −20 °C, respectively, for a maximum of three months. α and γ tocopherol were diluted in 100% dimethyl sulfoxide (DMSO) to a concentration of 1000 µM. These stock solutions were kept at 4 °C for a maximum of three months. Further dilutions were made using supplemented media, and the concentration of DMSO the cells were exposed to was lower than 0.8% DMSO. The crystal violet stain (0.5%) was prepared in a 50% methanol (99.9% pure). Destain solution for the crystal violet assay was prepared with 100% acetic acid diluted to 33% with demineralised water.

### 2.3. Cell Culture

The MCF-7 human epithelial breast adenocarcinoma cell line and the T47D human epithelial breast ductal carcinoma cell line were obtained from the America Type Culture Collection (ATCC, Manassas, VA, USA) and maintained in supplemented RPMI medium with 10% FCS. The OVCAR-3 human epithelial ovarian adenocarcinoma cell line (ATCC, Manassas, VA, USA) was maintained in RPMI medium supplemented with 20% FCS and 5 μg/mL insulin. The COV434 (ECACC 07071909) human ovarian granulosa cancer cell line was maintained in supplemented DMEM/F12 medium. Media in each 75 cm^2^ flask of cells were replaced every 2–3 days and each cell line was subcultured twice a week. Cells that had undergone fewer than 25 passages were used for all experiments when they were 80% confluent, and in the exponential growth phase.

### 2.4. Determination of MCF-7 Effective Concentration (EC) Values

MCF-7 cells (20,000 cells per well) were exposed to increasing concentrations of chemotherapeutics and tocopherols for 24 h and cell viability was examined in a crystal violet assay. The effective concentration that killed 50% and 25% of MCF-7 cells was calculated by a non-linear regression analysis performed using GraphPad Prism (Version 5.00, San Diego, CA, USA). The experiment was repeated on three separate occasions.

### 2.5. Effect of Dox, 4-Hydroperoxycyclophosphamide (4-Cyc), α or γ Tocopherol on ROS Generation

MCF-7, T47D, OVCAR-3 or COV434 cells (20,000 cells per well) were added to dark, clear bottom 96-well microplates for 24 h to adhere before adding each test agent to triplicate wells. Cells were exposed to 100 μL 10 μM DCFDA for 45 min at 37 °C in a humidified 5% CO_2_ incubator in the dark. The DCFDA solution was removed, and cells were exposed to 100 µL of chemotherapeutics or tocopherols (Table 1) for 24 h. Concentrations of chemotherapeutics and γToc were the effective concentrations that killed 25% of MCF-7 cells (EC25). Since a cytotoxic concentration of αToc was not determined, the highest concentration tested was selected for further examination. 

Controls were cells in medium only (background negative control), and cells exposed to low (12.5 μM) or high (50 μM) concentrations of TBHP (positive controls) [38], or 0.8% DMSO as a vehicle control for the tocopherols. Each experiment was repeated on three separate occasions (*n* = 3).

### 2.6. ROS Measurement by DCFDA Assay

The ROS production was detected by recording fluorescence immediately after addition of test agents (time 0), every hour for a 3 h incubation period, and after 24 h continuous incubation. Fluorescence was measured according to protocol described by Figueroa et al., [38]. Fluorescence readings were made using a plate spectrofluorometer (GloMax^®^ Explorer, Promega, Sydney, Australia). Relative fluorescence units (RFU) for each culture well were calculated by subtracting background readings (cells in media only), from all fluorescence values obtained from DCFDA loaded cells in media + test reagents. Each concentration of DCFDA and TBHP was examined in triplicate wells. Plates were sealed to maintain sterility during fluorescence readings and kept at 37 °C in a humidified 5% CO_2_ incubator in the dark between readings.

### 2.7. Crystal Violet (CV) Assay

After measurement of ROS, cell viability was determined using crystal violet (4-[(4-dimethylaminophenyl)-phenyl-methyl]-*N*,*N*-dimethyl-aniline) to stain DNA [39,40,41,42]. In short, 20,000 cells per well were cultured for 24 h to allow adherence, then loaded with DCFDA and exposed to test reagents. ROS were measured 0, 1, 2, 3 and 24 h after adding chemotherapeutics and tocopherols. Media containing test agents and non-adherent dead cells were removed, the cells were rinsed with PBS, and the PBS was replaced with 50 μL of crystal violet stain (0.5%) for 10 min. Cells were rinsed with demineralised water to remove any excess stain, then left to air-dry overnight. A total of 60 μL destain solution of 33% acetic acid was added for 10 min before absorbance was read at 570 nm with correction at 630 nm [41]. Linear correlations between optical density and cell number have been reported [41,42], therefore the numbers of viable cells remaining after exposure to test agents were determined by a comparison with a CV standard curve using densities of 0–80,000 cells per well (*R*^2^ = 0.99) generated for the same replicate experiment. Since CV stains DNA, the optical density values included contributions from any stained and adherent DNA, such as that included in condensed nuclei in the early stages of apoptosis or other forms of cell death, and adherent apoptotic bodies characteristic of the later stages of apoptosis.

### 2.8. DAPI Staining and Scoring of Cell Nuclei

6-diamidino-2-phenylindole (DAPI), a blue fluorescent dye, binds A–T-rich regions in dsDNA and has been used to visualise condensed, deformed or fragmented nuclei formed during both necrosis and apoptosis [8,43]. Early apoptosis is characterised by cell shrinkage and increased membrane permeability, which facilitates uptake of nuclear dyes such as DAPI. This causes condensed chromatin to appear as ‘bright’ dye-dense areas, whereas during late apoptosis the nucleus fragments and forms smaller apoptotic bodies. 

MCF-7, T47D, OVCAR-3 or COV434 cells (30,000 cells per well) were added to Nunc Lab-Tek II –CC2 chamber units (Promega, Sydney, Australia). After an initial 24 h adherence period to the glass microscope slide, cells were exposed to 300 µL of chemotherapeutics with or without tocopherols (Table 1) and incubated for another 24 h. The test reagents were removed, and the cells rinsed with PBS before fixation with 4% paraformaldehyde in PBS for 25 min at 4 °C. The cells were rinsed with PBS, then incubated with 1 µg/mL DAPI prepared in sterile PBS for 30 min in the dark at room temperature. After rinsing with PBS, cells were mounted in buffered glycerol and examined using an Olympus fluorescence microscope with filter Chroma 31,000 at excitation 340–380 nm, Dichroic 400 and emission 435–485 nm [44]. Four digital images of each well were taken at 20× magnification and the experiment was repeated on three separate occasions (*n* = 3) for each of the four cell types.

**Figure 1:**
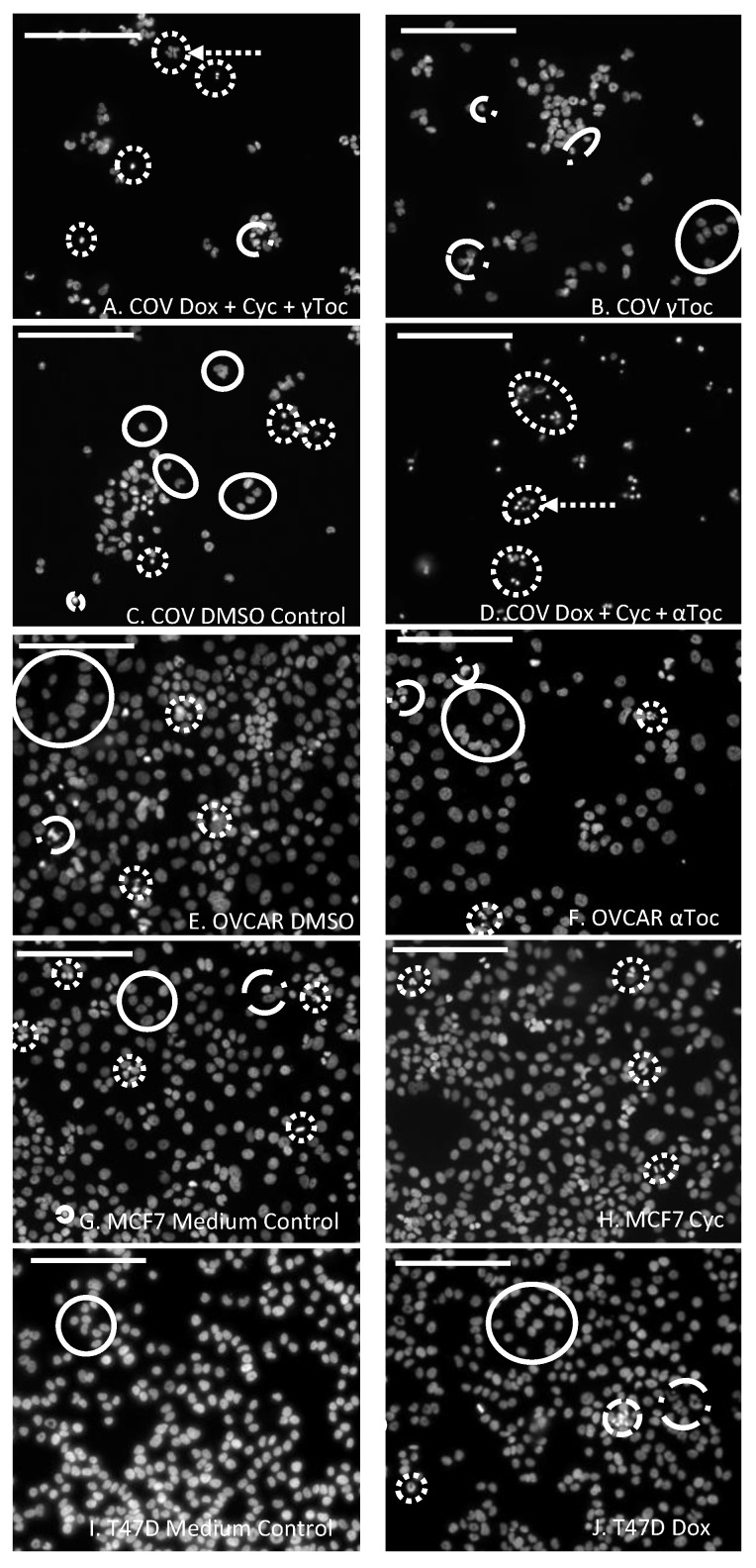
Cell nucleus 6-diamidino-2-phenylindole (DAPI) staining and scoring: COV434 (**A**–**D**), OVCAR (**E**,**F**), MCF7 (**G**,**H**) or T47D (**I*,*J**) cells adhered to the glass microscope slides for 24 h before exposure to doxorubicin (Dox, **J**) or 4-cyclophosphamide (Cyc, **H**) or α Tocopherol (αT, **F**) or γ Tocopherol (γT, **B**) or Dox + 4-Cyc + αToc (**D**) or Dox + 4-Cyc + γToc (**A**) or cell culture medium as a control for the chemotherapeutics (**G**,**I**), or 0.8% DMSO as a control for treatments containing tocopherols (**C**,**E**) were stained with DAPI before a fluorescence microscope was used to obtain digital images. The experiment was repeated on three separate occasions (*n* = 3) for each of the four cell types. Representative examples in each image shown of nuclei with normal morphology (solid circles), condensed DAPI-bright nuclei (dotted circles) or groups of apoptotic bodies (dotted circles and arrow) and nuclei with uncertain status (broken dash and dot circles). Scale bars 100 μm.

Scoring DAPI-stained nuclei in digital images is a subjective pastime. The scoring criteria were determined by reviewing published reports [45,46,47] and by observation of MCF7 images from the present study. Very small, bright (DAPI-intense) objects, that appeared as though the nucleus had fragmented into smaller apoptotic bodies (Figure 1A,D), or small very bright objects, usually with irregular shapes suggestive of condensed nuclei (Figure 1A,D,H,I), were collectively scored as condensed nuclei. Larger, relatively dull objects with regular spherical outlines (Figure 1E–G), or sometimes crescent-shaped outlines, particularly for the COV434 cells (Figure 1C), were scored as normal nuclei. Objects with irregular outlines that were smaller and brighter than normal nuclei were scored as uncertain. In some cases, they were only slightly larger than condensed nuclei, but if they had less DAPI (were duller), they were considered to have less condensed DNA and were classified as uncertain (Figure 1B,F). Groups of dull objects with a similar morphology to groups of apoptotic bodies were included in the condensed category (Figure 1A). Objects scored as uncertain were not clearly apoptotic or condensed nuclei, but neither were they unequivocally normal nuclei. Only complete nuclei or objects were included in the count and objects on the edges of the images were excluded.

Each image was allocated a code and deidentified, shuffled into a random order and scored blind. The scores were recorded onto each image before re-identifying and entering the numbers of condensed, uncertain or normal nuclei into a spreadsheet. The numbers of condensed nuclei (includes apoptotic bodies that were given a score of 1 for each group) were expressed as a percentage of the normal nuclei for each image (i.e., the uncertain nuclei were excluded from this calculation). There were three independent experiments, except for some cases (OVCAR and MCF7 medium control, MCF7 Dox + 4-Cyc + αToc, MCF7 Dox + 4-Cyc + γToc and MCF7 Dox) in which the experiment was repeated on four separate occasions. The effects of the chemotherapeutics and tocopherols on the nuclear morphology of MCF7, T47D and OVCAR cells were very similar, and hence representative examples have been shown in Figure 1. The mean ± stdev (*n* = 3 or 4) was calculated for the percentages of condensed nuclei. Data were subjected to 1Way ANOVA with Tukey post-hoc test.

### 2.9. Statistical Analysis

One-way ANOVA with Tukey HSD post-hoc tests were applied to the 24 h crystal violet, ROS and DAPI datasets, and a Two-way ANOVA with Bonferroni post-test was applied to the 24 h ROS control data using GraphPad Prism, GraphPad Software, San Diego, CA, USA). Two-way ANOVA with Bonferroni post-hoc tests were performed to examine the effect of 1–3 h exposure and reagent concentration on ROS production. These statistical analyses were performed using SPSS statistics software (V22.0 IBM, Armonk, NY, USA). Statistical significance was set at *p* ≤ 0.05. All experiments were performed as three independent replicates, and all data expressed as mean ± standard deviation.

## 3. Results

The numbers of DAPI-stained nuclei with normal regular morphology in the images of cell culture medium control wells were T47D (415 ± 68, Figure 1I) > MCF7 (312 ± 18 Figure 1G) > OVCAR (173 ± 32) > COV434 (53 ± 19) and there were similar numbers of nuclei with normal morphology in the 0.8% DMSO controls. The OVCAR cells were derived from ovarian surface epithelial cells, and the T47D and MCF7 cells were derived from mammary epithelial cells. The normal DAPI-stained nuclei of these three cell lines in control wells were a similar size and morphology, with regular round or oval shapes (Figure 1E,G,I). The COV434 cells were derived from a granulosa cell tumour, and their nuclei were much smaller and often shaped like a croissant (crescent, Figure 1C). DAPI-intense ‘bright’ objects were observed in two forms: a relatively low number of apoptotic bodies (Figure 1A,D) and much higher numbers of what appeared to be small, irregularly shaped condensed nuclei (Figure 1A,H). Some nuclei were clearly not ‘normal’, but neither were they bright nor small enough to qualify as ‘condensed’. These were labelled ‘uncertain’ and were frequently observed in the COV434 cell line (8.9%, Figure 1B), but were found less frequently in the MCF7 (2.4%), OVCAR (1.4%) and T47D cells (1.2%).

**Figure 2:**
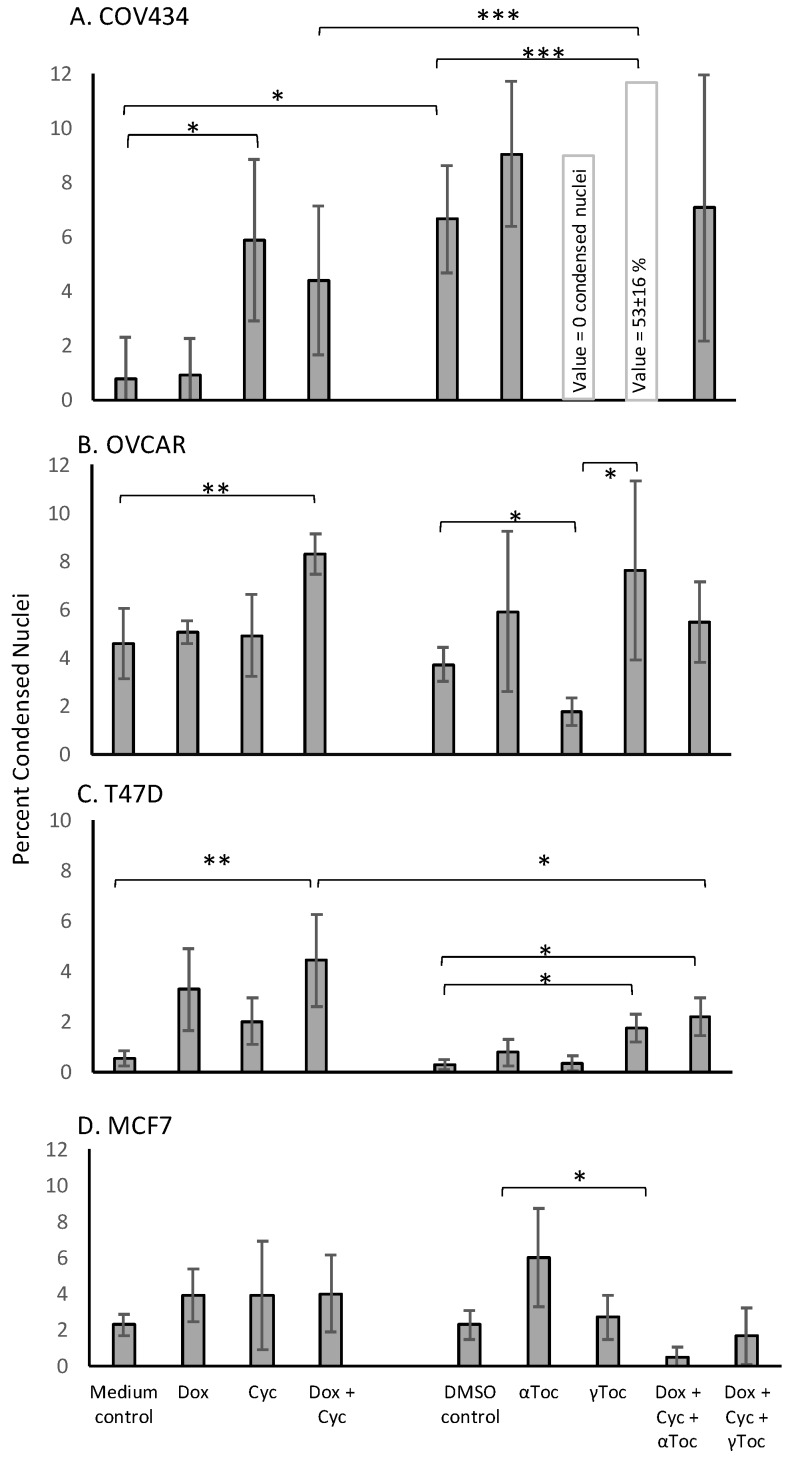
DAPI-stained condensed cell nuclei: COV434 (**A**), OVCAR (**B**), T47D (**C**) or MCF7 (**D**) cells adhered to glass microscope slides for 24 h before a 24 h exposure to doxorubicin (Dox) or 4-cyclophosphamide (Cyc) or α Tocopherol (αToc) or γ Tocopherol (γToc) or combinations of these. Cell culture medium was a control for the chemotherapeutics and 0.8% DMSO in culture medium was a control for tocopherols. Cells were stained with DAPI before a fluorescence microscope was used to capture digital images. The numbers of normal and condensed (includes groups of apoptotic bodies) nuclei were scored in each image, and the condensed nuclei expressed as a percentage. The experiment was repeated on three separate occasions and the mean ± stdev of percentages is shown. Data were subjected to 1 Way ANOVA with a Tukey post-test * *p* < 0.05, ** *p* < 0.01, *** *p* < 0.001.

Although the numbers of nuclei with normal morphology were similar in cell culture medium and in medium containing 0.8% DMSO in all four cell lines (representative examples shown in Figure 1), a 24 h culture in 0.8% DMSO caused significantly more condensed COV434 nuclei than medium control (*p* < 0.05, Figure 2A), whereas the percentages of condensed nuclei were similar in media and DMSO controls in the other three cell lines (Figure 2). 4-Cyc significantly increased the percentage of condensed COV434 nuclei (*p* < 0.05) compared to the cell culture medium control. The COV434 cell nuclei in control cell culture media were smaller than the nuclei of the other three cell lines, but exposure to the combination of Dox + 4-Cyc + αToc (Figure 1D) caused the nuclei which most resembled ‘normal’ to become even smaller. Nuclei exposed to Dox + 4-Cyc + αToc were difficult to score because it was not clear if they should be placed in the condensed or uncertain categories. It was clear, however, that there were very few normal nuclei; only 9.6 ± 1.5 normal (Figure S1A) and 12.3 ± 7.8 condensed nuclei (53 ± 16%, Figure 2A) per image, as opposed to 59.5 ± 3.5 normal nuclei in the COV434 DMSO control images (Figure 1C). Although exposure to αToc alone resulted in the same proportions of condensed nuclei as in the DMSO control, the addition of αToc to the chemotherapeutics significantly increased the percentage of condensed nuclei compared to the DMSO control and to Dox + 4-Cyc (*p* < 0.001, Figure 2A). Even though the COV434 DAPI-stained nuclei were difficult to score, it was still clear that αToc increased the cytotoxic activity of Dox + 4-Cyc.

Conversely, γToc reduced the background levels of the condensed COV434 nuclei to zero (Figure 2A) and the addition of γToc to the chemotherapeutics maintained the number of normal nuclei at 48 ± 9 (Figure 1B and Figure S1A), hence there were no statistical differences between the percentages of condensed nuclei in DMSO control and Dox + 4-Cyc + γToc treated COV434 cells (Figure 2A). 

Neither of the chemotherapeutics affected the percentages of condensed MCF7 nuclei (Figure 2B). MCF7 cells exposed to αToc for 24 h had 375 ± 101 normal nuclei and 21 ± 4 condensed nuclei (6 ± 2.7%, Figure 2B), whereas exposure to Dox + 4-Cyc + αToc resulted in only 90 ± 20 normal nuclei (Figure S1D) and 0.48 ± 0.6% condensed nuclei (Figure 2B). This reduction in condensed nuclei occurred in the context of cell loss indicative of cell death earlier in the 24 h exposure and was unlikely to be caused by αToc protecting MCF7 against the cytotoxic effects of Dox + 4-Cyc. MCF7 cells cultured in DMSO control conditions or exposed to Dox + 4-Cyc + γToc were like the COV434 cells in that there were no significant differences in the numbers of normal nuclei, nor the percentages of condensed nuclei. 

Dox + 4-Cyc increased the number of condensed T47D nuclei (*p* < 0.01) compared to the medium control, and the addition of each of the tocopherols to Dox + 4-Cyc increased the percentages of condensed nuclei compared to the DMSO control (*p* < 0.05, Figure 2C). Addition of the tocopherols to the chemotherapeutics also increased the numbers of normal nuclei; there were 253 ± 13 normal nuclei after exposure to Dox + 4-Cyc, but 503 ± 290 and 650 ± 73 normal nuclei after exposure to the chemotherapeutics combined with αToc and γToc, respectively (Figure S1C). 

The combination of Dox + 4-Cyc also increased the number of condensed OVCAR nuclei (*p* < 0.01, Figure 2D). Although γToc reduced the number of condensed nuclei compared to the DMSO control (*p* < 0.05), when the tocopherols were combined with Dox + 4-Cyc neither affected the proportions of condensed (Figure 2D) or normal nuclei (Figure S1B).

Crystal violet stains DNA. The proportion of condensed nuclei in all cases except one (COV434 Dox + 4-Cyc + αToc) was lower than 10% (Figure 2). It is therefore reasonable to assume that at least 90% of the crystal violet staining was indicative of viable cells containing nuclei with normal morphologies. Exposure to 0.8% DMSO, the vehicle for both tocopherols, did not affect the amount of crystal violet staining in any cell line, for example, there were 33,837 ± 1642 T47D cells after 24 h in medium control and 37,897 ± 495 in control medium containing 0.8% DMSO (Figure S1). Although 20,000 cells were initially seeded into the 96 well plates, after a total of 48 h in control conditions there were fewer COV434 cells than the other three cell lines—22,948 ± 1567 COV434 cells per well (Figure S1).

**Figure 3:**
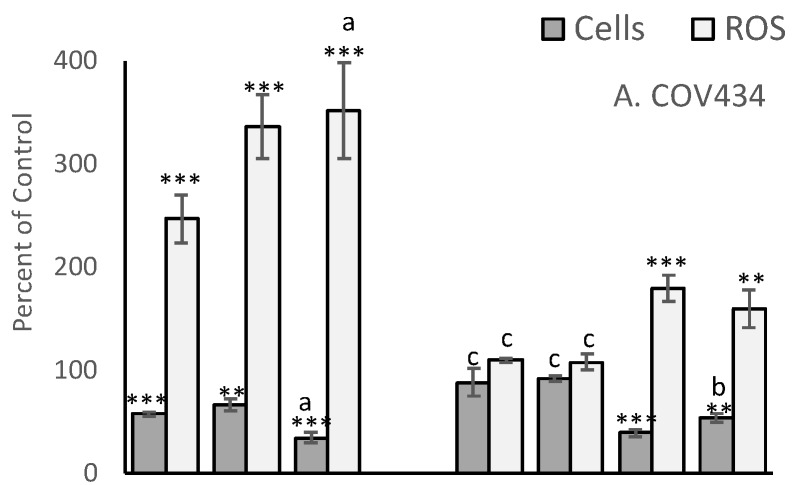

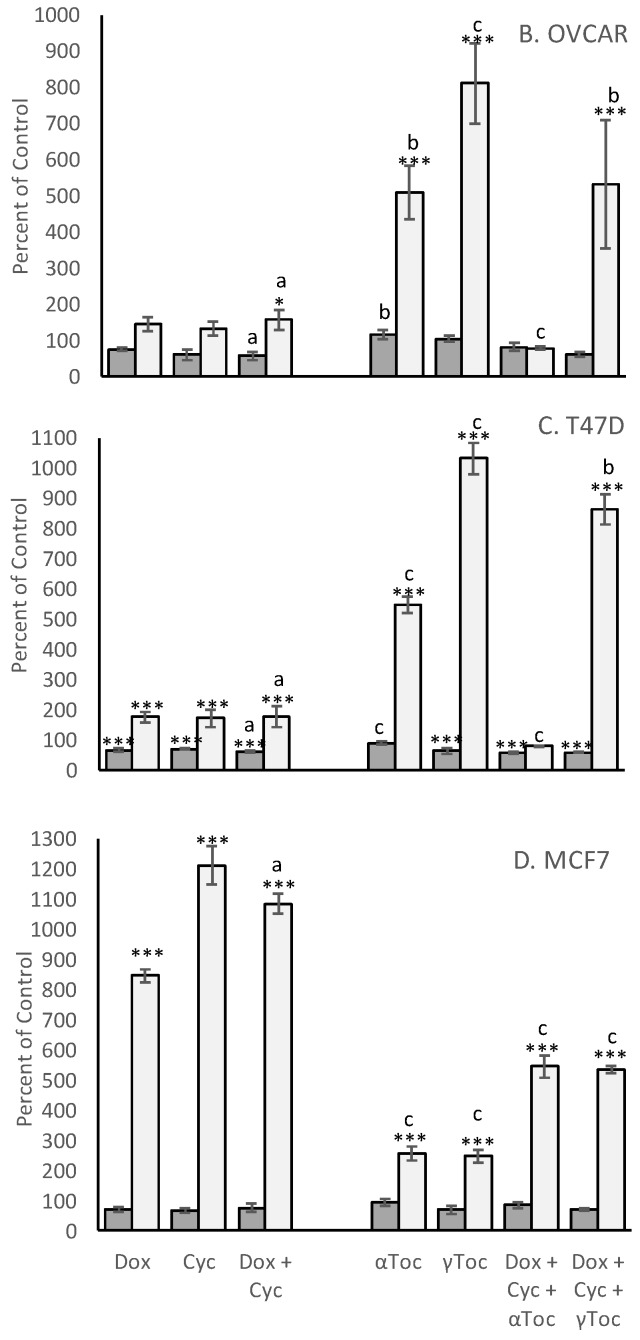
Effect of chemotherapeutics and tocopherols on cell viability and reactive oxygen species (ROS). Human COV434 (**A**), OVCAR (**B**), T47D (**C**) and MCF7 (**D**) cells were cultured for 24 h before being loaded with DCFDA and exposed to Doxorubicin (Dox) or 4-Cyclophosphamide (Cyc) or α Tocopherol (αToc) or γ Tocopherol (γToc), or combinations of these, for 24 h in triplicate wells. ROS were measured before measuring the number of adherent cells per well in a crystal violet assay (Cells). The average cell number, or ROS, obtained from triplicate wells, was expressed as a percentage of control for the same experimental replicate. Controls were culture medium, or culture medium containing DMSO which was the vehicle for tocopherols. Data are shown as mean ± stdev of percentages obtained in three independent experiments (*n* = 3). The original ‘Cells’ or ‘ROS’ data (i.e., not percentages) were subjected to 1 Way ANOVA with a Tukey post-test. Within either Cells or ROS, significant difference from control * *p* > 0.05, ** *p* > 001, *** *p* > 0.001, or a v b *p* > 0.05, b v c *p* < 0.01, a v c *p* > 0.001.

The MCF-7 derived EC25 values for 4-Cyc reduced viable cell numbers by approximately 25% in the present study. MCF-7 viable cell numbers were reduced to 68 ± 9% of control, T47D to 71 ± 2%, COV434 to 66 ± 6% and OVCAR to 61 ± 15% (Figure 3). Cell line sensitivity to the cytotoxic effects of Dox was slightly different. COV434 cells were the most sensitive (57 ± 2%), whereas the three epithelial cell lines had similar sensitivities, ranging from 67 ± 6% (T47D) to 75 ± 5% (OVCAR). The combination of the EC25 value for 4-Cyc with the EC25 value for Dox was expected to cause death of 50% of the cells, but was less cytotoxic to MCF-7 (76 ± 14% of control) and T47D (61 ± 4%) but more cytotoxic to COV434 cells (34 ± 5% of control). For OVCAR cells, the cytotoxicity caused by the two chemotherapeutics was additive; the combination reduced viable OVCAR cell numbers to 57 ± 11% of control. 

A cytotoxic dose of αToc was not found, and the relatively high non-cytotoxic concentration of αToc used in the present study was not cytotoxic to any cell line (Figure 3). The MCF7-derived EC25 value of γToc reduced T47D cell viability to 64 ± 9% (*p* < 0.01, Figure 3C) and MCF7 cells to 70 ± 14% of DMSO control but had no effect on the viability of COV434 or OVCAR cells (Figure 3). γToc interacted with the chemotherapeutics and COV434 cells such that Dox + 4-Cyc reduced viable COV434 cell numbers to 34 ± 5% of medium control (*p* < 0.001), but when γToc was added to the combined chemotherapeutics COV434 cell viability was reduced to 54 ± 4% of the DMSO control (*p* < 0.01). Hence, when compared to Dox + 4-Cyc, γToc conferred a significant protective effect against the cytotoxicity caused by the chemotherapeutics (*p* < 0.05). However, γToc did not affect the cytotoxicity of the combined chemotherapeutics in the other three cell lines.

**Figure 4:**
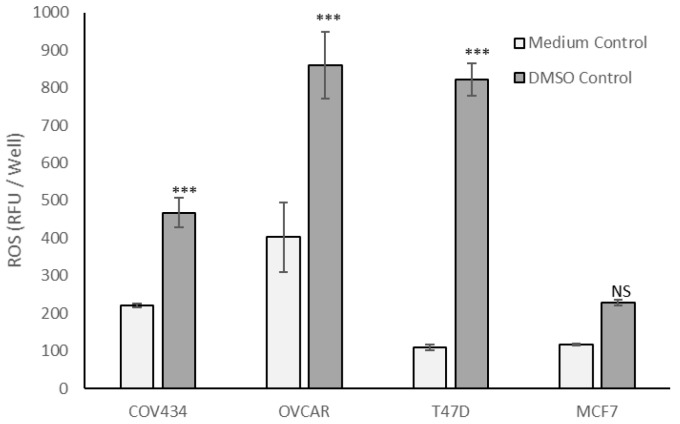
Reactive oxygen species (ROS) generation under in vitro control conditions. Cells were cultured for 24 h before being loaded with DCFDA, then cultured for 24 h in either cell culture medium (Medium Control) or cell culture medium containing 0.8% DMSO (DMSO Control). Relative Fluorescent Units (RFU per culture well) indicative of reactive oxygen species (ROS) generated by the cells in that well were measured using a 96-well plate spectrofluorometer. Data are shown as mean ± stdev of three independent experiments (*n* = 3). The RFU per well values were analysed by 2-Way ANOVA with Bonferroni post-test. Within each cell line, significant difference between two controls: NS not significant, *** *p* > 0.001.

Although 0.8% DMSO, the vehicle control for tocopherols, had no effect on cell viability (Figure S1), COV434, OVCAR and T47D cells cultured in 0.8% DMSO generated significantly more ROS than in culture medium (*p* < 0.001, Figure 4), whereas MCF7 cells generated similar amounts of ROS in the two control media (Figure 4).

After 24 h exposure, 4-Cyc caused all four cell lines to generate more ROS than Dox (Figure 3). The addition of the MCF7-derived EC25 4-Cyc to the EC25 Dox did not double ROS compared to each chemotherapeutic alone, but ROS levels after exposure to the combined chemotherapeutics were always higher than after exposure to 4-Cyc alone. Neither αToc nor γToc affected ROS production by COV434, OVCAR or T47D cells, but it was surprising that each tocopherol significantly increased ROS in MCF7 cells (*p* < 0.001 Figure 3D). The addition of tocopherols to Dox + 4-Cyc was unable to prevent ROS generation by MCF7, T47D or COV434 cells, but αToc reduced and γToc completely prevented the chemotherapeutic-stimulated increase in ROS.

**Figure 5:**
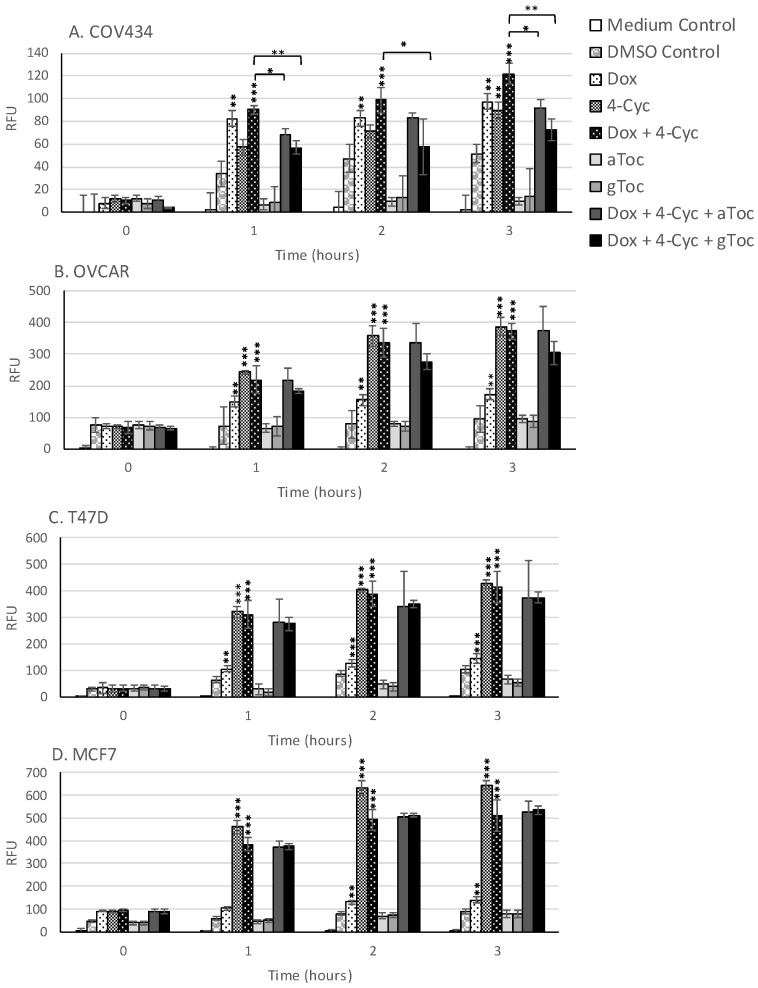
Effect of 3 h exposure to chemotherapeutics and tocopherols on ROS production. (**A**) COV434, (**B**) OVCAR, (**C**) T47D, and (**D**) MCF-7 cells were loaded with DCFDA then exposed to Doxorubicin (Dox), 4-Cyclophosphamide (4-Cyc), α Tocopherol (aToc) or γ Tocopherol (gToc) or combinations of these for 3 h at concentrations that reduced MCF-7 viability by 25% (EC25). Fluorescence was read every hour for 3 h. Means ± SD of three independent experiments are shown. Relative fluorescent units (RFU) were subjected to Two-way ANOVA with Bonferroni post-hoc test * *p* ≤ 0.05, ** *p* ≤ 0.01, *** *p* ≤ 0.001 significant difference from same exposure control, bars show significant difference compared to combination of Dox and 4-Cyc at same exposure.

Although ROS levels did not change in cell culture medium, ROS levels increased in the cell culture medium containing 0.8% DMSO during the first 3 h of culture (Figure 5). Acute, time-dependent, significant increases in ROS levels were detected in MCF-7, T47D and OVCAR-3 cells during the first 3 h exposure to the MCF-7 EC25 value (21.23 µM) of 4-Cyc (Figure 5). Dox caused a lower, but still significant increase in ROS production by the same cell lines. In COV434 granulosa cells, ROS levels increased after 1 h exposure to Dox. Unlike the other cell lines, 4-Cyc and Dox stimulated the same amount of ROS during the first 3 h; the amount of ROS generated by 4-Cyc was lower than in the other three cell lines (Figure 5). α and γToc did not stimulate ROS generation after 1, 2 or 3 h in any of the cell lines. 

The combination of Dox + 4-Cyc also caused time-dependent, significant increases in ROS within the first 3 h of exposure (Figure 5). The addition of either tocopherol to the combination of chemotherapeutics had no effect on acute ROS generation by three of the cell lines, but significantly reduced ROS generation by COV434 cells. γToc was more effective than αToc at reducing Dox + 4-Cyc generated ROS in COV434 cells within the first 3 h of exposure.

## 4. Discussion

This is the first study to examine the effect of a clinically relevant combination of the chemotherapeutics Dox and 4-Cyc on cytotoxicity and ROS production by human breast and ovarian cell lines in vitro. Our finding that γToc reduced chemotherapeutic-generated ROS production by transformed ovarian granulosa COV434 and epithelial adenocarcinoma OVCAR cells, but not by breast cancer cells, indicates the potential to develop antioxidant γToc as an adjunct treatment to reduce the adverse effects of chemotherapeutic-stimulated ROS on proliferating ovarian granulosa cells.

DAPI and CV staining showed that there were similar numbers of viable cells in cell culture medium and culture medium containing 0.8% DMSO, but there were approximately 33% fewer viable COV434 cells in the culture medium than in the other three cell lines. The cell doubling times have been reported as COV434 24 h [48] to 36 h [49], MCF7 30 h [50], T47D 39 h [50] and OVCAR 48 h [51]. If doubling times were the explanation for the difference in viable cell numbers there should have been fewer OVCAR than COV434 cells, because OVCAR are reported as having the slowest doubling times. Although the ECACC recommends growing COV434 cells in high glucose DMEM, Tsai-Turton et al. (2007, [52]) used high glucose DMEM/F12, and we repeated their method because primary-derived human granulosa cells are commonly cultured in the same medium [53,54,55]. In the present study, however, the pH indicator phenol red was omitted to avoid interference with ROS quantification. Phenol red is a weak estrogen and COV434 cells respond to estrogen by proliferating. We speculate that our estrogen-depleted control culture medium resulted in lower levels of COV434 proliferation. The addition of 0.8% DMSO to this culture medium did not change the numbers of DAPI-stained COV434 nuclei with normal morphology, nor the numbers of viable cells quantified in our crystal violet assay, but significantly increased ROS and the percentages of COV434 condensed nuclei. It is likely that, if COV434 cells were cultured in 0.8% DMSO for longer periods of time (than 24 h), the numbers of viable cells would decrease to reflect the increase in ROS and condensed cell nuclei.

Breast cancer patients are commonly administered an infusion of Dox intravenously (60 mg/m^2^) then an infusion of cyclophosphamide (600 mg/m^2^) [56,57], and different types of breast cancer have additional agents added to their treatment regimens (e.g., addition of paclitaxel to four cycles of Dox and cyclophosphamide [58,59], but breast cancer is not treated with either agent alone. In early animal studies, the combination of Dox and cyclophosphamide was therapeutically potentiating against four different murine mammary tumour lines compared to Dox as a single agent [60] and this was attributed to different cytotoxic mechanisms of action. However, we have been unable to locate any in vitro or human in vivo studies that compared Dox + cyclophosphamide to either Dox or cyclophosphamide alone. 

The chemotherapeutics (without tocopherols) stimulated ROS in all four cell lines, reduced cell viability in COV434 and T47D cells, and increased the percentage of condensed cell nuclei in COV434, OVCAR and T47D cells, hence chemotherapeutic-stimulated ROS production was associated with cell damage when measured using DAPI or CV staining. 4-Cyc induced significant ROS generation in all cell lines within 1 h, and this increased 3- to 10-fold after 24 h exposure. Our data support previous reports that increases in ROS mediate 4-Cyc-induced apoptosis in different types of cells [17,18]. Dox on the other hand, did not generate ROS as quickly as 4-Cyc, and in three of the cell lines Dox generated fewer ROS than 4-Cyc after 24 h exposure. ROS production in H9c2 cardiac muscle cells exposed for 1 h to 10 µM Dox was four times higher than in the medium control [61]. In the present study, the lower 1.21 µM concentration of Dox that killed 25% of MCF-7 cells was compared with Tan et al. (2010) [61], and this suggests that higher amounts of ROS are produced with increasing concentrations of Dox. COV434 cells exposed to 50 µM 4-Cyc for 2 h, or 1 µM for 6 h, significantly increased the production of ROS [52]. Although the concentration of DCFDA used in this study was 100 times higher than the one used in our experiments, and higher DCFDA concentrations can be toxic and contribute to ROS generation [38], the amount of ROS generated by COV434 cells after 24 h exposure to 21 µM 4-Cyc in our study was in broad agreement with the previous study [52].

The four cell lines displayed different sensitivities to the cytotoxic and ROS-inducing activities of the test agents. Several factors affect cell line responses in vitro [62]. One factor is the cell doubling time, because some chemotherapeutics are phase-specific agents, which means that only cells that are passing through the relevant cell cycle phase when the drug is present are killed [63,64,65]. Because cells that are in a different cell cycle phase are not targeted by the phase-specific agent, a single of dose of the drug may only kill a fixed fraction of cells and multiple doses may be needed to eradicate the tumour [66]. ‘Fractional kill’ predicts a strong correlation between proliferation rate and drug sensitivity. Neither Dox or cyclophosphamide are considered cell cycle phase-specific drugs, but they have been known to preferentially target more metabolically active cells [23], and Fan et al. [67] found that Dox inhibited the growth of HepG2 cells by induction of G2/M cell cycle arrest. It’s therefore possible that cell lines with longer doubling times might require a longer exposure time to Dox and 4-Cyc in vitro for cytotoxic effects. However, MCF7 cells have the shortest reported doubling time (30 h, [50]) but were the least sensitive to the cytotoxic effects of the combined chemotherapeutics. Previously, MCF7 and ovarian granulosa KGN cells were exposed to 5 µM Dox or 4-Cyc for 24 h then cultured for a further 48 h or exposed for 72 h continuously in culture [68]. There was no difference in cytotoxicity between these two exposure regimens; >80% of both cell lines died within the first 24 h. In the present study, then, the differing proliferation rates of the four cell lines do not appear to explain the data.

Another factor that affects in vitro responses to test agents is the origin and phenotype of the cell line. The MCF-7 and T47D cell lines were isolated from a pleural effusion of patients with breast carcinoma [69,70]. MCF-7 cells maintain several of the functional characteristics of differentiated mammary epithelium, including the expression of estrogen receptors [71], which means that control proliferation rates may have been reduced in our phenol red-free system. Similarly, the T47D line expresses receptors for estradiol, progesterone and other steroid hormones [70]. The COV434 cell line was derived from a solid primary human ovarian granulosa cell carcinoma but is a good in vitro model for normal healthy granulosa cells because the cell line maintains many of the functional characteristics required for follicle growth and development [72]. These include the expression of estrogen receptors and a steroidogenic pathway that enables COV434 cells to synthesise steroid hormones such as estrogen and progesterone. Steroidogenesis generates ROS, and human steroidogenic ovarian cells have relatively high levels of intracellular antioxidants which include Vitamin E in humans [73,74]. The OVCAR-3 cell line also synthesizes steroid hormones and was obtained from a patient who was administered a combination of Dox, cyclophosphamide and cisplatin to treat an epithelial adenocarcinoma of the ovary. Eight months later, her ascites fluid-containing ovarian adenocarcinoma cells were injected into nude mice, and the resulting tumours were disaggregated and used to generate the OVCAR-3 cell line. These cells are resistant to clinically relevant concentrations of doxorubicin, although the effects of 4-Cyc have not been reported [57]. In the present study, the MCF7-derived EC25 value of Dox reduced viable OVCAR cells to 75 ± 5% of control, which suggests that OVCAR have a resistance to Dox comparable to MCF7 cells, whereas the same concentration of Dox reduced COV434 cell viability to 57 ± 2% of control. The combination of the EC25 Dox with the EC25 4-Cyc was additive towards OVCAR cells (57 ± 2% of control) but did not increase cytotoxicity towards MCF7 (76 ± 14%) or T47D cells. Although the combined chemotherapeutics were not more cytotoxic to the two breast cancer cell lines than either agent alone, there were marked increases in chemotherapeutic-stimulated ROS, to 1413 ± 230% (T47D) and 1085 ± 31% (MCF7) of control. If the chemotherapeutics also stimulate a 10-fold increase in ROS in vivo, perhaps it is accompanied by a more impressive increase in cytotoxicity than occurred in our in vitro system. The combined chemotherapeutics displayed synergistic cytotoxicity towards the granulosa-derived COV434 cells, and the reduction in viable cells to 34 ± 5% of control was accompanied by a 770 ± 84% increase in ROS. In this study, the viability of the granulosa tumour-derived COV434 cell line was more sensitive to the clinically relevant combination of Dox + 4-Cyc than the two breast cancer cell lines. More experiments are needed to determine if primary-derived physiologically normal granulosa cells display the same sensitivity, but since immortalised cancer-derived cell lines are generally more robust and resistant to cytotoxic agents than mortal primary-derived cells, we predict that proliferating follicular granulosa cells will be more sensitive to Dox + 4-Cyc than breast cancer cells.

Cancer-derived cell lines are different from tumours in vivo, and cell lines and tumours are different from physiologically normal, healthy cells. Tumour-derived COV434 granulosa cells, however, retain features of physiologically normal granulosa cells, and the ovarian epithelial OVCAR cells are different from the mammary epithelial cells because they, like normal granulosa and COV434 cells, retain a steroidogenic pathway. The steroidogenic pathway and associated intracellular antioxidants imply differences in redox status between the two ovarian and two breast cancer cell lines. This frames our hypothesis that antioxidant tocopherols might reduce chemotherapeutic-induced ROS and their associated damage in granulosa-like steroidogenic cells, whilst maintaining the anti-cancer cytotoxicity of the chemotherapeutics against breast cancer cells. Therefore, one of the aims of this study was to discover the effect of αToc and γToc on ROS induced by exposure to the combination of Dox + 4-Cyc. 

In our earlier study, we did not find a concentration of αToc that killed MCF7 breast cancer cells, and selected the highest concentration tested. In the present study, this relatively high concentration of αToc alone did not kill any cell type compared to the DMSO control, had no significant effect on the proportions of condensed nuclei, and did not affect ROS generation in three of the four cell lines, although both tocopherols stimulated MCF7 ROS generation somewhere between 3 and 24 h of exposure. The addition of αToc to Dox + 4-Cyc for 24 h decreased T47D cell viability while increasing condensed nuclei, and significantly increased ROS in all four cell lines. The COV434 cells differed from the other three cell lines in that αToc reduced chemotherapeutic-stimulated ROS in the first 3 h of exposure, but in the subsequent 3–24 h of exposure the combination of Dox + 4-Cyc + αToc caused significantly more ROS, condensed nuclei and loss of viable COV434 cells than Dox + 4-Cyc without αToc, and additionally caused COV434 nuclei to shrink. Steroid hormones and tocopherols are lipophilic and miscible with cell membrane lipid bilayers. The chemotherapeutics Dox and 4-Cyc are soluble in water, and this caused us to speculate that if the relatively high, non-cytotoxic concentration of αToc used in this study were to affect the fluidity and plasticity of the cell membranes, it may have improved chemotherapeutics’ access to the interior of the cell in some way. Another difference between COV434 cells and the other three cell lines is their size; COV434 cells are smaller and probably have a higher surface area to volume ratio, which would increase the importance of membrane changes and chemotherapeutic uptake relative to the other three cell lines. Our finding that αToc increased the cytotoxicity of Dox + 4-Cyc against the COV434 granulosa tumour cell line, in an estrogen-depleted in vitro system, provides a rationale for further investigation.

A 24 h exposure to the concentration of γToc that killed 25% of MCF7 cells in a previous study had no effect on condensed nuclei but significantly increased ROS and killed approximately 25% of the two breast cancer cell lines. In contrast, the same 24 h exposure to the MCF7 EC25 value of γToc did not kill the two ovarian cell lines and the lower percentages of condensed nuclei corresponded to the higher numbers of viable cells. Although 24 h exposure to γToc stimulated MCF7 ROS, it had no effect on ROS in any other cell line. It is interesting that, in the two ovarian cell lines, γToc alone not only prevented an increase in ROS levels but no condensed nuclei at all were observed in COV434 cells, and there were significantly fewer condensed OVCAR nuclei than in the DMSO control (*p* < 0.05). These differences between the two steroidogenic cell lines may reflect their different sizes, or the different steroid hormones they synthesise.

Twenty-four hours exposure to Dox + 4-Cyc (without γToc) increased ROS, reduced cell viability and generally increased the percentage of condensed nuclei in all four cell lines. The addition of γToc to Dox + 4-Cyc nearly halved chemotherapeutic-induced ROS in COV434 cells within 1 h and maintained this inhibition for 3 h but had no effect on ROS in the other three cell lines. This early inhibition of ROS in COV434 cells may have been the reason there was no increase in condensed nuclei after 24 h, and why there were significantly more viable cells after 24 h exposure to Dox + 4-Cyc + γToc than after exposure to Dox + 4-Cyc without γToc. The different responses to the combination of chemotherapeutics and γToc displayed by COV434 and the other three cell lines are unlikely to be explained by antioxidant or REDOX status and are more likely to be related to the interaction of γToc with apoptotic pathways within the cells. MCF7 and T47D cells have different apoptotic pathways, which may have resulted in significant increases in condensed nuclei in T47D but not MCF7 cells. OVCAR are resistant to Dox, and probably to 4-Cyc too, and this may account for the maintenance of viable cell numbers and lack of increase in condensed cell nuclei, despite significant increases in ROS. Nevertheless, the finding that γToc reduced COV434 ROS for 3 h and prevented nucleus condensation and loss of cell viability for 24 h, whilst stimulating ROS and nucleus condensation and decreasing T47D breast cancer cell viability during a 24 h exposure, supports our hypothesis: γToc reduced chemotherapeutic-induced ROS and associated damage in granulosa-like steroidogenic cells, whilst maintaining the anti-cancer cytotoxicity of the chemotherapeutics against breast cancer cells.

## 5. Conclusions

The clinical efficacy of the combined regimen of Dox and cyclophosphamide against breast cancer in vivo is probably related to their combined cytotoxicity as well as their ability to increase ROS production. One way to improve existing anti-cancer treatments is to reduce off-target adverse effects without reducing efficacy against breast cancer. In the present study, the addition of γToc to Dox and 4-Cyc differentially and specifically reduced ROS levels after only 1 h in the ovarian granulosa cell line COV434 and maintained the percentages of condensed nuclei indicative of cell damage at the same levels as the DMSO control in the two ovarian cell lines expressing steroidogenic pathways, whereas γToc increased cell damage caused by the chemotherapeutics in the breast cancer T47D cell line. If the protective effects of γToc in the presence of Dox + 4-Cyc can be repeated in normal, non-cancerous, primary-derived granulosa cells and the chemotherapeutic enhancing effects of γToc can be demonstrated in primary-derived breast cancer tumour cells, this will confirm the potential for antioxidant γToc to be developed as an adjunct to existing breast cancer chemotherapy, which will improve fertility preservation for premenopausal breast cancer patients. 

## Figures and Tables

**Table 1:** 24 h MCF-7-derived EC25 chemotherapeutics and tocopherols values.

**Single Agents**	**Concentrations (µM)**
Dox	1.21
4-Cyc	21.23
αToc	100
γToc	35.1
**Combined Agents**	**Concentrations (µM)**
Dox + 4-Cyc	1.21 (Dox) + 21.23 (4-Cyc)
Dox + 4-Cyc + αToc	1.21 (Dox) + 21.23 (4-Cyc) + 100 (αToc)
Dox + 4-Cyc + γToc	1.21 (Dox) + 21.23 (4-Cyc) + 35.1 (γToc)

Dox—Doxorubicin, 4-Cyc—4-hydroperoxycyclophosphamide, αToc—α-Tocopherol, γToc—γ-Tocopherol.

## Supplementary Materials

The following are available online at https://www.mdpi.com/2076-3921/9/1/51/s1, **Figure S1**: Comparison of DAPI and Crystal Violet Datasets: The numbers of human COV434 (**A**), MCF7 (**B**), T47D (**C**) and OVCAR (**D**) cells per well that were determined using a crystal violet assay were divided by 100 to allow comparison with the DAPI dataset on the same axis. The crystal violet values show mean ± stdev (*n* = 3). The DAPI values are the sum of nuclei with ‘condensed’ + ‘uncertain’ + normal morphologies scored in images from three replicate experiments. Each group of apoptotic bodies were assumed to have been formed by the fragmentation of a single nucleus and were therefore given a score of ‘1’. These were added to the numbers of small, irregularly shaped DAPI-dense nuclei. Nuclei classified as ‘uncertain’ formed 8.9% (COV434), 2.4% (MCF7), 1.4% (OVCAR) and 1.2% (T47D) of the total numbers of nuclei in all assessed images, whereas the normal nuclei formed 85% (COV434), 95% (MCF7), 94% (OVCAR) and 97.2% (T47D). DAPI values shown as the mean ± stdev (*n* = 3). The crystal violet cell data were subjected to 1Way ANOVA with a Tukey post-test. Significant difference from control. ** *p* > 001, *** *p* > 0.001, or a v b *p* > 0.05, a v c *p* > 0.001. **Figure S2**: Images of DAPI-stained cell nuclei obtained using an Olympus AX70 fluorescence microscope at ×20 magnification after cells were exposed to chemotherapeutics and tocopherols for 24 h. Dox—doxorubicin, Cyc—cyclophosphamide, Toc—tocopherol.

## References

[1] Fan L.M., Li J.-M. (2014). Evaluation of methods of detecting cell reactive oxygen species production for drug screening and cell cycle studies. J. Pharmacol. Toxicol. Methods.

[2] Gomes A., Fernandes E., Lima J.L. (2005). Fluorescence probes used for detection of reactive oxygen species. J. Biochem. Biophys. Methods.

[3] Valko M., Leibfritz D., Moncol J., Cronin M.T., Mazur M., Telser J. (2007). Free radicals and antioxidants in normal physiological functions and human disease. Int. J. Biochem. Cell Biol..

[4] Droge W. (2002). Free radicals in the physiological control of cell function. Physiol. Rev..

[5] Doroshow J.H. (1986). Role of hydrogen peroxide and hydroxyl radical formation in the killing of Ehrlich tumor cells by anticancer quinones. Proc. Natl. Acad. Sci. USA.

[6] Gutteridge J.M., Halliwell B. (2010). Antioxidants: Molecules, medicines, and myths. Biochem. Biophys. Res. Commun..

[7] Halliwell B. (1991). Drug antioxidant effects. Drugs.

[8] Nogueira V., Hay N. (2013). Molecular pathways: Reactive oxygen species homeostasis in cancer cells and implications for cancer therapy. Clin. Cancer Res..

[9] Bray J., Sludden J., Griffin M., Cole M., Verrill M., Jamieson D., Boddy A. (2010). Influence of pharmacogenetics on response and toxicity in breast cancer patients treated with doxorubicin and cyclophosphamide. Br. J. Cancer.

[10] Joerger M., Huitema A.D.R., Richel D.J., Dittrich C., Pavlidis N., Briasoulis E., Vermorken J.B., Strocchi E., Martoni A., Sorio R. (2007). Population Pharmacokinetics and Pharmacodynamics of Doxorubicin and Cyclophosphamide in Breast Cancer Patients. Clin. Pharmacokinet..

[11] Nabholtz J.M., Falkson C., Campos D., Szanto J., Martin M., Chan S., Pienkowski T., Zaluski J., Pinter T., Krzakowski M. (2003). Docetaxel and doxorubicin compared with doxorubicin and cyclophosphamide as first-line chemotherapy for metastatic breast cancer: Results of a randomized, multicenter, phase III trial. J. Clin. Oncol..

[12] Tewey K., Rowe T., Yang L., Halligan B., Liu L.-F. (1984). Adriamycin-induced DNA damage mediated by mammalian DNA topoisomerase II. Science.

[13] Gewirtz D. (1999). A critical evaluation of the mechanisms of action proposed for the antitumor effects of the anthracycline antibiotics adriamycin and daunorubicin. Biochem. Pharmacol..

[14] Mizutani H., Tada-Oikawa S., Hiraku Y., Kojima M., Kawanishi S. (2005). Mechanism of apoptosis induced by doxorubicin through the generation of hydrogen peroxide. Life Sci..

[15] Emadi A., Jones R.J., Brodsky R.A. (2009). Cyclophosphamide and cancer: Golden anniversary. Nat. Rev. Clin. Oncol..

[16] Boddy A.V., Yule S.M. (2000). Metabolism and Pharmacokinetics of Oxazaphosphorines. Clin. Pharmacokinet..

[17] Liu F., Li X.-L., Lin T., He D.-W., Wei G.-H., Liu J.-H., Li L.-S. (2012). The cyclophosphamide metabolite, acrolein, induces cytoskeletal changes and oxidative stress in Sertoli cells. Mol. Biol. Rep..

[18] Mythili Y., Sudharsan P.T., Selvakumar E., Varalakshmi P. (2004). Protective effect of dl-α-lipoic acid on cyclophosphamide induced oxidative cardiac injury. Chem. Biol. Interact..

[19] Legha S.S., Benjamin R.S., Mackay B., Ewer M., Wallace S., Valdivieso M., Rasmussen S.L., Blumenschein G.R., Freireich E.J. (1982). Reduction of doxorubicin cardiotoxicity by prolonged continuous intravenous infusion. Ann. Intern. Med..

[20] Ozer H., Cowens J.W., Colvin M., Nussbaum-Blumenson A., Sheedy D. (1982). In vitro effects of 4-hydroperoxycyclophosphamide on human immunoregulatory T subset function. I. Selective effects on lymphocyte function in TB cell collaboration. J. Exp. Med..

[21] Meirow D., Lewis H., Nugent D., Epstein M. (1999). Subclinical depletion of primordial follicular reserve in mice treated with cyclophosphamide: Clinical importance and proposed accurate investigative tool. Hum. Reprod..

[22] Meirow D., Biederman H., Anderson R.A., Wallace W.H.B. (2010). Toxicity of chemotherapy and radiation on female reproduction. Clin. Obstet. Gynecol..

[23] Morgan S., Anderson R., Gourley C., Wallace W., Spears N. (2012). How do chemotherapeutic agents damage the ovary?. Hum. Reprod. Update.

[24] Petrillo S.K., Desmeules P., Truong T.-Q., Devine P.J. (2011). Detection of DNA damage in oocytes of small ovarian follicles following phosphoramide mustard exposures of cultured rodent ovaries in vitro. Toxicol. Appl. Pharmacol..

[25] Oktem O., Oktay K. (2007). A novel ovarian xenografting model to characterize the impact of chemotherapy agents on human primordial follicle reserve. Cancer Res..

[26] Oktem O., Oktay K. (2007). Quantitative assessment of the impact of chemotherapy on ovarian follicle reserve and stromal function. Cancer.

[27] Jurisicova A., Lee H., D’Estaing S., Tilly J., Perez G. (2006). Molecular requirements for doxorubicin-mediated death in murine oocytes. Cell Death Differ..

[28] Perez G.I., Knudson C.M., Leykin L., Korsmeyer S.J., Tilly J.L. (1997). Apoptosis-associated signaling pathways are required for chemotherapy-mediated female germ cell destruction. Nat. Med..

[29] Soleimani R., Heytens E., Darzynkiewicz Z., Oktay K. (2011). Mechanisms of chemotherapy-induced human ovarian aging: Double strand DNA breaks and microvascular compromise. Aging.

[30] Iqubal M.A., Khan M., Kumar P., Kumar A., Ajai K. (2014). Role of Vitamin E in Prevention of Oral Cancer: A Review. J. Clin. Diagn. Res..

[31] Brigelius-Flohe R., Kelly F., Salonen J., Neuzil J., Zingg J., Azzi A. (2002). The European perspective on vitamin E: Current knowledge and future research. Am. J. Clin. Nutr..

[32] Myers C.E., McGuire W.P., Liss R.H., Ifrim I., Grotzinger K., Young R.C. (1977). Adriamycin: The role of lipid peroxidation in cardiac toxicity and tumor response. Science.

[33] Smolarek A., Suh N. (2011). Chemopreventive Activity of Vitamin E in Breast Cancer: A Focus on gamma- and delta-Tocopherol. Nutrients.

[34] Klein E.A., Thompson I.M., Tangen C.M., Crowley J.J., Lucia M.S., Goodman P.J., Minasian L., Ford L.G., Parnes H.L., Gaziano J.M. (2011). Vitamin E and the Risk of Prostate Cancer: Updated Results of The Selenium and Vitamin E Cancer Prevention Trial (SELECT). JAMA.

[35] Gopalan A., Yu W., Jiang Q., Jang Y., Sanders B.G., Kline K. (2012). Involvement of de novo ceramide synthesis in gamma-tocopherol and gamma-tocotrienol-induced apoptosis in human breast cancer cells. Mol. Nutr. Food Res..

[36] Lee H.J., Ju J., Paul S., So J.-Y., DeCastro A., Smolarek A., Lee M.-J., Yang C.S., Newmark H.L., Suh N. (2009). Mixed Tocopherols Prevent Mammary Tumorigenesis by Inhibiting Estrogen Action and Activating PPAR-γ. Clin. Cancer Res..

[37] Constantinou C.A., Papas A., Constantinou A.I. (2008). Vitamin E and cancer: An insight into the anticancer activities of vitamin E isomers and analogs. Int. J. Cancer.

[38] Figueroa D., Asaduzzaman M., Young F. (2018). Real time monitoring and quantification of reactive oxygen species in breast cancer cell line MCF-7 by 2′,7′–dichlorofluorescin diacetate (DCFDA) assay. J. Pharmacol. Toxicol. Methods.

[39] Yang Y.-I., Jung D.-W., Bai D.-G., Yoo G.-S., Choi J.-K. (2001). Counterion-dye staining method for DNAin agarose gels using crystal violet and methyl orange. Electrophoresis.

[40] Berry J.M., Huebner E., Butler M. (1996). The crystal violet nuclei staining technique leads to anomalous results in monitoring mammalian cell cultures. Cytotechnology.

[41] Vega-Avila E., Pugsley M.K. (2011). An Overview of Colorimetric Assay Methods Used to Assess Survival or Proliferation of Mammalian Cells. Proc. West. Pharmacol. Soc..

[42] Reid K.J., Lang K., Froscio S., Humpage A.J., Young F.M. (2015). Undifferentiated murine embryonic stem cells used to model the effects of the blue–green algal toxin cylindrospermopsin on preimplantation embryonic cell proliferation. Toxicon.

[43] Atale N., Gupta S., Yadav U.C., Rani V. (2014). Cell-death assessment by fluorescent and nonfluorescent cytosolic and nuclear staining techniques. J. Microsc..

[44] Anderson W.F., Rosenberg P.S., Prat A., Perou C.M., Sherman M.E. (2014). How Many Etiological Subtypes of Breast Cancer: Two, Three, Four, Or More?. JNCI J. Natl. Cancer Inst..

[45] Mathieu C., Jozan S., Mazars P., Come M.G., Moisand A., Valette A. (1995). Density-Dependent Induction of Apoptosis by Transforming Growth Factor-beta1 in a Human Ovarian Carcinoma Cell Line. Exp. Cell Res..

[46] Kolb R.H., Greer P.M., Cao P.T., Cowan K.H., Yan Y. (2012). ERK1/2 Signaling Plays an Important Role in Topoisomerase II Poison-Induced G2/M Checkpoint Activation. PLoS ONE.

[47] Im J.Y., Park H., Kang K.W., Choi W.S., Kim H.S. (2008). Modulation of cell cycles and apoptosis by apicidin in estrogen receptor (ER)-positive and-negative human breast cancer cells. Chem. Biol. Interact..

[48] Cowley G.S., Weir B.A., Vazquez F., Tamayo P., Scott J.A., Rusin S., East-Seletsky A., Ali L.D., Gerath W.F., Pantel S.E. (2014). Parallel genome-scale loss of function screens in 216 cancer cell lines for the identification of context-specific genetic dependencies. Sci. Data.

[49] Verga Falzacappa C., Mangialardo C., Patriarca V., Bucci B., Amendola D., Raffa S., Torrisi M.R., Silvestrini G., Ballanti P., Moriggi G. (2009). Thyroid hormones induce cell proliferation and survival in ovarian granulosa cells COV434. J. Cell. Physiol..

[50] Dougherty M.K., Schumaker L.M., Jordan V.C., Welshons W.V., Curran E.M., Ellis M.J., El-Ashry D. (2004). Estrogen receptor expression and sensitivity to paclitaxel in breast cancer. Cancer Biol. Ther..

[51] Hamilton T.C., Young R.C., McKoy W.M., Grotzinger K.R., Green J.A., Chu E.W., Whang-Peng J., Rogan A.M., Green W.R., Ozols R.F. (1983). Characterization of a human ovarian carcinoma cell line (NIH: OVCAR-3) with androgen and estrogen receptors. Cancer Res..

[52] Tsai-Turton M., Luong B.T., Tan Y., Luderer U. (2007). Cyclophosphamide-Induced Apoptosis in COV434 Human Granulosa Cells Involves Oxidative Stress and Glutathione Depletion. Toxicol. Sci..

[53] Young F.M., Micklem J., Humpage A.R. (2008). Effects of blue-green algal toxin cylindrospermopsin (CYN) on human granulosa cells in vitro. Reprod. Toxicol..

[54] Young F.M., Zebian D., Froscio S., Humpage A. (2012). Cylindrospermopsin, a blue-green algal toxin, inhibited human luteinised granulosa cell protein synthesis in vitro. Toxicol. In Vitro.

[55] Edwards V., Benkendorff K., Young F. (2012). Novel marine compounds selectively induce apoptosis in female reproductive cancer cells but not in primary-derived human granulosa cells. Mar. Drugs.

[56] Dees E.C., O’Reilly S., Goodman S.N., Sartorius S., Levine M.A., Jones R.J., Grochow L.B., Donehower R.C., Fetting J.H. (2000). A prospective pharmacologic evaluation of age-related toxicity of adjuvant chemotherapy in women with breast cancer. Cancer Investig..

[57] Jones S.E., Savin M.A., Holmes F.A., O’Shaughnessy J.A., Blum J.L., Vukelja S., McIntyre K.J., Pippen J.E., Bordelon J.H., Kirby R. (2006). Phase III trial comparing doxorubicin plus cyclophosphamide with docetaxel plus cyclophosphamide as adjuvant therapy for operable breast cancer. J. Clin. Oncol..

[58] Yardley D., Arrowsmith E., Daniel B., Eakle J., Brufsky A., Drosick D., Kudrik F., Bosserman L., Keaton M., Goble S. (2017). TITAN: Phase III study of doxorubicin/cyclophosphamide followed by ixabepilone or paclitaxel in early-stage triple-negative breast cancer. Breast Cancer Res. Treat..

[59] Henderson I.C., Berry D.A., Demetri G.D., Cirrincione C.T., Goldstein L.J., Martino S., Ingle J.N., Cooper M.R., Hayes D.F., Tkaczuk K.H. (2003). Improved outcomes from adding sequential paclitaxel but not from escalating doxorubicin dose in an adjuvant chemotherapy regimen for patients with node-positive primary breast cancer. J. Clin. Oncol..

[60] Corbett T., Griswold D., Mayo J., Laster W., Schabel F. (1975). Cyclophosphamide-adriamycin combination chemotherapy of transplantable murine tumors. Cancer Res..

[61] Tan X., Wang D.-B., Lu X., Wei H., Zhu R., Zhu S.-S., Jiang H., Yang Z.-J. (2010). Doxorubicin induces apoptosis in H9c2 cardiomyocytes: Role of overexpressed eukaryotic translation initiation factor 5A. Biol. Pharm. Bull..

[62] Nunzio M.D., Valli V., Tomás-Cobos L., Tomás-Chisbert T., Murgui-Bosch L., Danesi F., Bordoni A. (2017). Is cytotoxicity a determinant of the different in vitro and in vivo effects of bioactives?. BMC Complement. Altern. Med..

[63] Gascoigne K.E., Taylor S.S. (2009). How do anti-mitotic drugs kill cancer cells?. J. Cell Sci..

[64] Shi J., Orth J.D., Mitchison T. (2008). Cell type variation in responses to antimitotic drugs that target microtubules and kinesin-5. Cancer Res..

[65] Orth J.D., Tang Y., Shi J., Loy C.T., Amendt C., Wilm C., Zenke F.T., Mitchison T.J. (2008). Quantitative live imaging of cancer and normal cells treated with Kinesin-5 inhibitors indicates significant differences in phenotypic responses and cell fate. Mol. Cancer Ther..

[66] Berenbaum M. (1972). In vivo determination of the fractional kill of human tumor cells by chemotherapeutic agents. Cancer Chemother. Rep..

[67] Fan C., Zheng W., Fu X., Li X., Wong Y.-S., Chen T. (2014). Strategy to enhance the therapeutic effect of doxorubicin in human hepatocellular carcinoma by selenocystine, a synergistic agent that regulates the ROS-mediated signaling. Oncotarget.

[68] Figueroa D., Asaduzzaman M., Young F. (2019). Effect of Chemotherapeutics and Tocopherols on MCF-7 Breast Adenocarcinoma and KGN Ovarian Carcinoma Cell Lines In Vitro. BioMed Res. Int..

[69] Soule H., Vazquez J., Long A., Albert S., Brennan M. (1973). A human cell line from a pleural effusion derived from a breast carcinoma. J. Natl. Cancer Inst..

[70] Keydar I., Chen L., Karby S., Weiss F., Delarea J., Radu M., Chaitcik S., Brenner H. (1979). Establishment and characterization of a cell line of human breast carcinoma origin. Eur. J. Cancer (1965).

[71] Huguet E.L., McMahon J.A., McMahon A.P., Bicknell R., Harris A.L. (1994). Differential expression of human Wnt genes 2, 3, 4, and 7B in human breast cell lines and normal and disease states of human breast tissue. Cancer Res..

[72] Zhang H., Vollmer M., De Geyter M., Litzistorf Y., Ladewig A., Dürrenberger M., Guggenheim R., Miny P., Holzgreve W., De Geyter C. (2000). Characterization of an immortalized human granulosa cell line (COV434). Mol. Hum. Reprod..

[73] Young F.M., Luderer W.B., Rodgers R.J. (1995). The antioxidant beta-carotene prevents covalent cross-linking between cholesterol side-chain cleavage cytochrome P450 and its electron donor, adrenodoxin, in bovine luteal cells. Mol. Cell. Endocrinol..

[74] Rodgers R.J., Lavranos T.C., Rodgers H.F., Young F.M., Vella C.A. (1995). The physiology of the ovary: Maturation of ovarian granulosa cells and a novel role for antioxidants in the corpus luteum. J. Steroid Biochem. Mol. Biol..

