# Supplementary material for: Gamma Tocopherol Reduced Chemotherapeutic-Induced ROS in an Ovarian Granulosa Cell Line, But Not in Breast Cancer Cell Lines In Vitro
Source: Antioxidants (Basel). 2020 Jan 7;9(1):51. doi: 10.3390/antiox9010051 (PMC7023168; doi:10.3390/antiox9010051)
Supplement: Supplementary file 1 [file antioxidants-09-00051-s001.pdf]

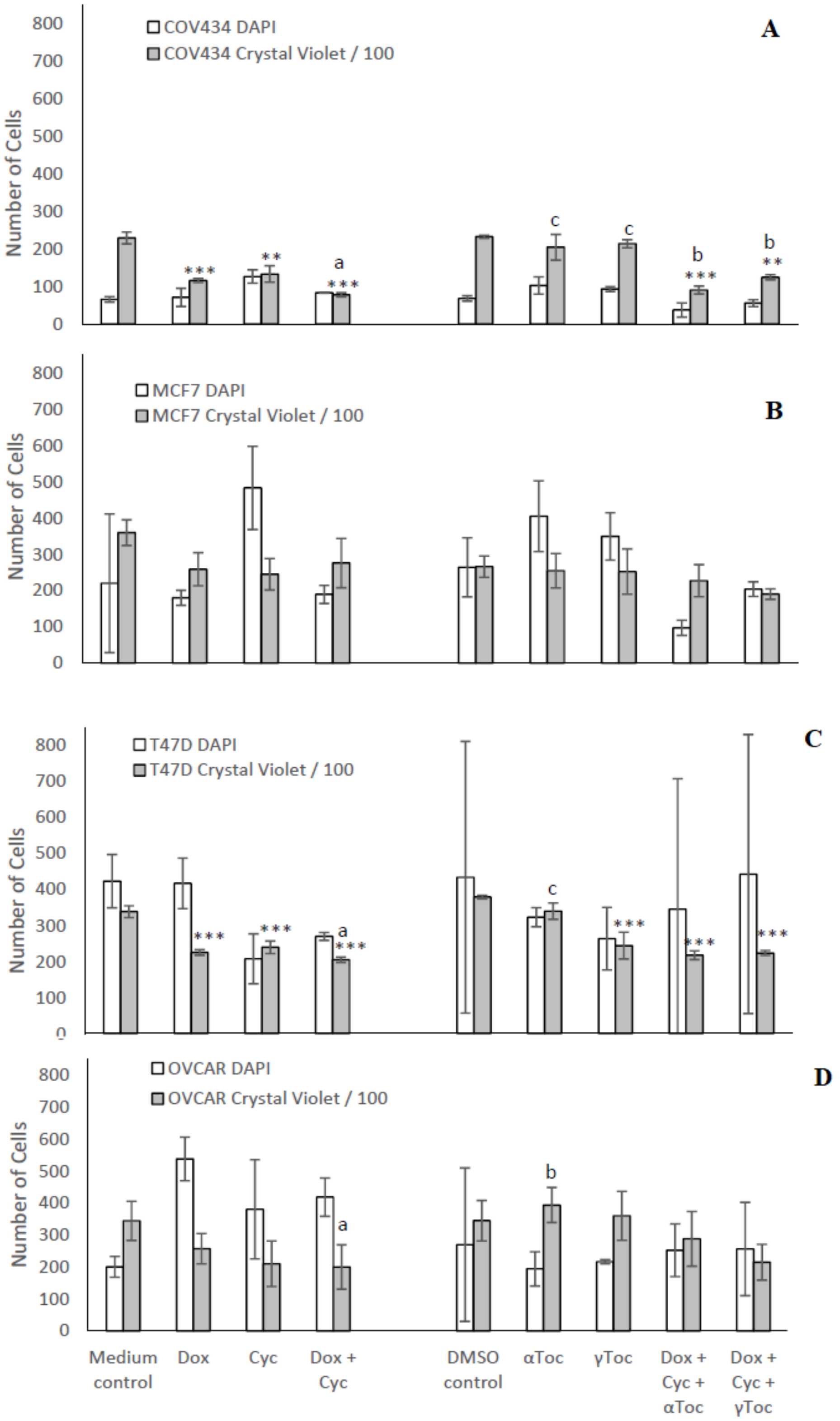

Figure S1: Comparison of DAPI and Crystal Violet Datasets: The numbers of human COV434 (**A**), MCF7 (**B**), T47D (**C**) and OVCAR (**D**) cells per well that were determined using a crystal violet assay were divided by 100 to allow comparison with the DAPI dataset on the same axis. The crystal violet values show mean ± stdev (*n* = 3). The DAPI values are the sum of nuclei with 'condensed' + 'uncertain' + normal morphologies scored in images from three replicate experiments. Each group of apoptotic bodies were assumed to have been formed by the fragmentation of a single nucleus and

were therefore given a score of '1'. These were added to the numbers of small, irregularly shaped DAPI-dense nuclei. Nuclei classified as 'uncertain' formed 8.9% (COV434), 2.4% (MCF7), 1.4% (OVCAR) and 1.2% (T47D) of the total numbers of nuclei in all assessed images, whereas the normal nuclei formed 85% (COV434), 95% (MCF7), 94% (OVCAR) and 97.2% (T47D). DAPI values shown as the mean ± stdev (n = 3). The crystal violet cell data were subjected to 1Way ANOVA with a Tukey post-test. Significant difference from control. ** $p > 001$, *** $p > 0.001$, or a v b $p > 0.05$, a v c $p > 0.001$.

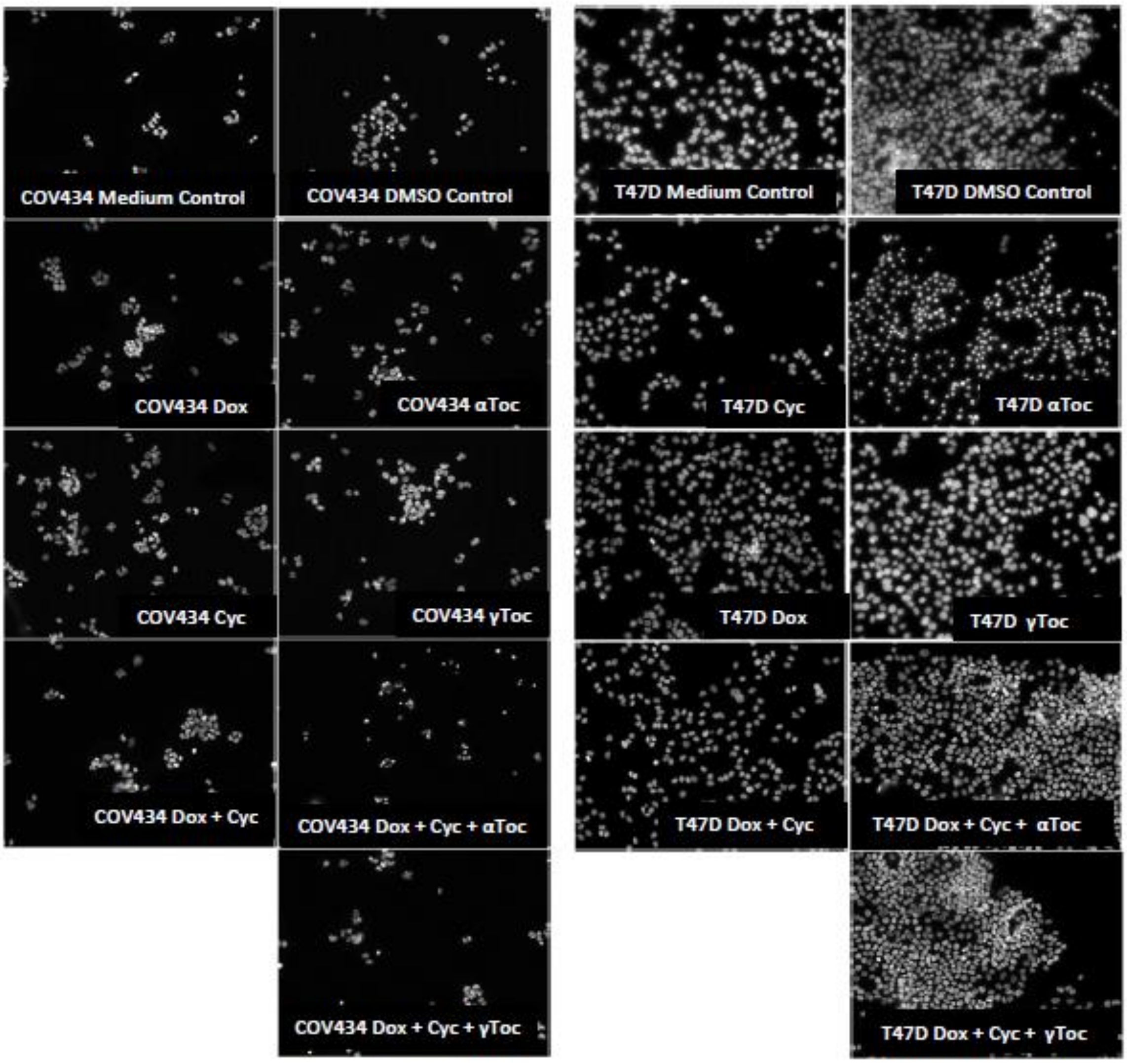

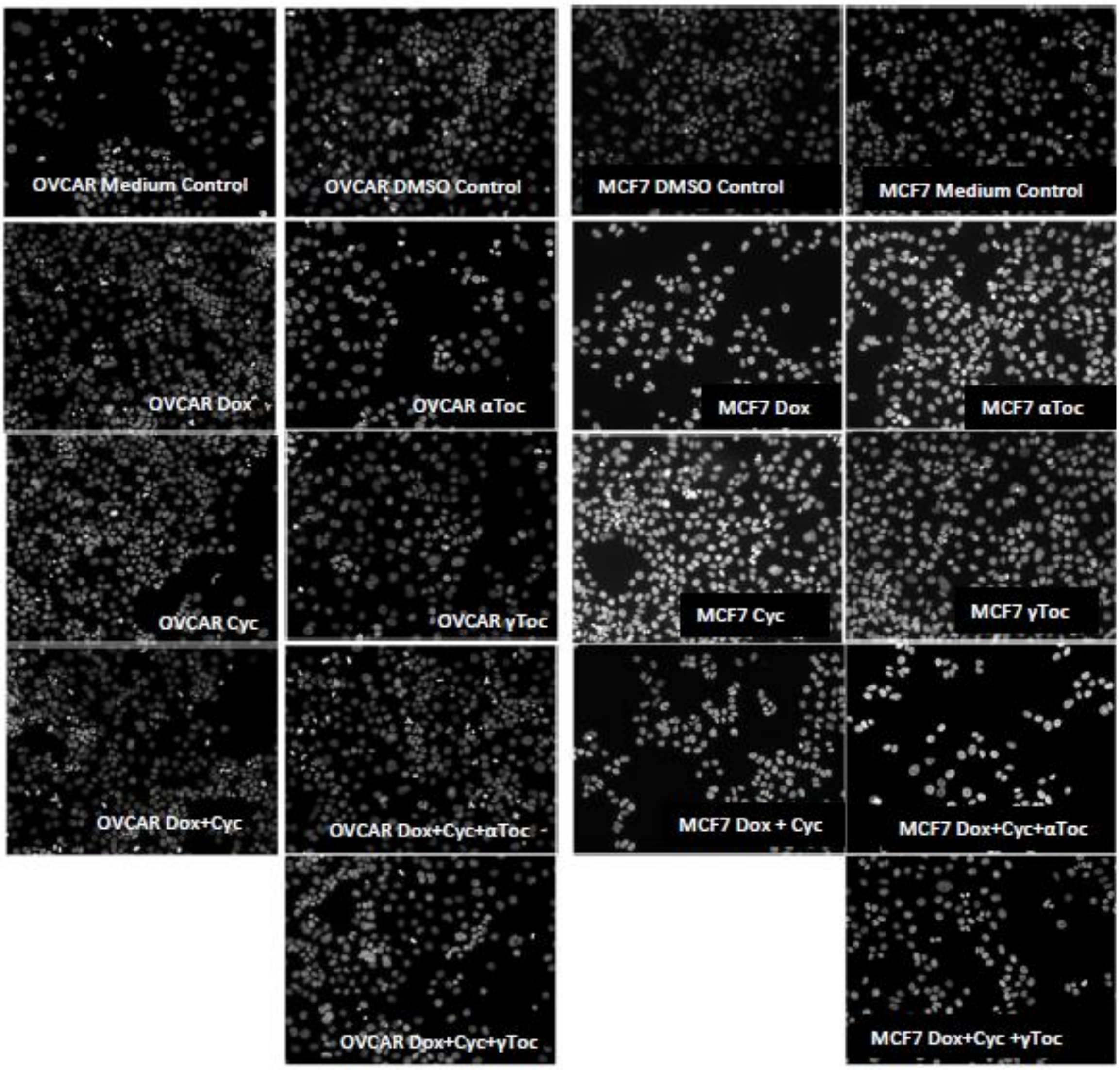

Figure S2: Images of DAPI-stained cell nuclei obtained using an Olympus AX70 fluorescence microscope at ×20 magnification after cells were exposed to chemotherapeutics and tocopherols for 24 h. Dox—doxorubicin, Cyc—cyclophosphamide, Toc—tocopherol.
